# The Role of DNA Barcodes in Understanding and Conservation of Mammal Diversity in Southeast Asia

**DOI:** 10.1371/journal.pone.0012575

**Published:** 2010-09-03

**Authors:** Charles M. Francis, Alex V. Borisenko, Natalia V. Ivanova, Judith L. Eger, Burton K. Lim, Antonio Guillén-Servent, Sergei V. Kruskop, Iain Mackie, Paul D. N. Hebert

**Affiliations:** 1 Canadian Wildlife Service, Environment Canada, Ottawa, Ontario, Canada; 2 Biodiversity Institute of Ontario, University of Guelph, Guelph, Ontario, Canada; 3 Department of Natural History, Royal Ontario Museum, Toronto, Ontario, Canada; 4 Instituto de Ecología A.C., Xalapa, Veracruz, México; 5 Zoological Museum, Moscow State University, Moscow, Russia; 6 Aberdeen Centre for Environmental Sustainability, University of Aberdeen, Aberdeen, United Kingdom; Montreal Botanical Garden, Canada

## Abstract

**Background:**

Southeast Asia is recognized as a region of very high biodiversity, much of which is currently at risk due to habitat loss and other threats. However, many aspects of this diversity, even for relatively well-known groups such as mammals, are poorly known, limiting ability to develop conservation plans. This study examines the value of DNA barcodes, sequences of the mitochondrial COI gene, to enhance understanding of mammalian diversity in the region and hence to aid conservation planning.

**Methodology and Principal Findings:**

DNA barcodes were obtained from nearly 1900 specimens representing 165 recognized species of bats. All morphologically or acoustically distinct species, based on classical taxonomy, could be discriminated with DNA barcodes except four closely allied species pairs. Many currently recognized species contained multiple barcode lineages, often with deep divergence suggesting unrecognized species. In addition, most widespread species showed substantial genetic differentiation across their distributions. Our results suggest that mammal species richness within the region may be underestimated by at least 50%, and there are higher levels of endemism and greater intra-specific population structure than previously recognized.

**Conclusions:**

DNA barcodes can aid conservation and research by assisting field workers in identifying species, by helping taxonomists determine species groups needing more detailed analysis, and by facilitating the recognition of the appropriate units and scales for conservation planning.

## Introduction

Southeast Asia has been identified as one of the world's biodiversity hotspots based on both plant and animal diversity [1]. Nearly 500 species of mammals and 1250 species of birds are currently recognized in mainland Southeast Asia [2], [3]. The numbers are nearly doubled if the archipelagos of Indonesia and the Philippines are included [4], [5], representing about 20% of the global totals for both groups. Moreover, at least for mammals, recent rates of species discovery suggest that true species richness is much higher than currently recognized. Reeder *et al*. [6] found that an average of 223 new species of mammals have been described per decade worldwide since 1758, with the rate increasing over time, suggesting that many more species await description. In Southeast Asia, six new species of ungulates have been described since 1992 [7] and some well known taxa, including the orang-utan (*Pongo pygmaeus*) and the clouded leopard (*Neofelis nebulosa*), have been found to represent more than one species (e.g., [8], [9]). In the same period since 1992, more than 50 new species of small mammals, including bats, rodents, and insectivores, have been described [10]. Because many of these newly identified taxa are geographically restricted, they may require special conservation measures relative to more widespread species that were described earlier.

Unfortunately, much of this diversity is now threatened and in need of urgent conservation action. One-quarter of global mammalian species diversity is threatened with extinction and half of the species have declining populations [11]. The situation for terrestrial mammals in Asia is particularly dire, because much of the native habitat has been heavily disturbed or lost and many species are being overharvested. More than 30% of vertebrate species in Asia are considered at risk (Near Threatened or higher); another 20% are considered data deficient so their status cannot be determined [12]. The conservation status of other groups of animals in the region is much less known, but, given the rates of habitat loss, many species are likely to be at risk.

Although they represent only a small fraction of total biodiversity, mammals and birds are often used for conservation planning on the assumption that their protection will conserve key habitat for many other taxa, and because their distribution and taxonomy are better known than most other taxa. Conservation of biodiversity in Southeast Asia requires a variety of measures including a network of protected areas sufficient to contain viable populations of as many species as possible. While the selection of key sites for biodiversity protection is now constrained by the availability of intact habitats, information on the distribution of species remains important for the selection of new protected areas and for determining and prioritizing conservation actions within existing ones.

Although mammals are better known than many other taxa, many gaps remain in our knowledge of their distribution and taxonomy in Southeast Asia that need resolution to enable effective conservation planning. Most species of birds in the region are well described (although new species are still being discovered) and can be identified at a distance through plumage traits or songs [3]. In contrast, although a number of field guides now exist for mammals in the region (e.g., [2], [13], [14]), only the larger species are readily recognized without capture and, even for them, taxonomy may be uncertain. For smaller mammals, careful examination of prepared specimens and comparison with reference material in museums is often required to confirm identifications using standard morphological approaches. This can be a particular challenge for field workers in Southeast Asia because critical reference material, such as type specimens, is scattered in the world's museums.

Since its initial proposal as a tool for rapid identification of species [15], DNA barcoding has gained considerable validation. Among terrestrial vertebrates, this approach has been shown to be effective in the identification of amphibians [16], North American birds [17], [18], Neotropical birds [19] and Neotropical small mammals [20], [21]. For North American birds, about 94% of currently recognized species could be uniquely identified by barcodes, while the remaining 6% could be identified to within one of two or more closely related species [18]; similar results were obtained for birds in Argentina [19]. For bats in Guyana, all 87 currently recognized species could be uniquely identified by DNA barcodes [20]. DNA barcodes are also proving to be a useful tool for identifying genetically distinct units worthy of more intense taxonomic study. For North American and Argentinean birds as well as Guyanese bats, about 10% of species sampled showed deep intra-specific divergences in DNA barcodes that may indicate previously unrecognized species or at least genetically divergent populations worth considering as distinct for the purposes of conservation planning [18], [20].

In this paper, we examine the value of DNA barcodes for enhancing our knowledge of the distribution and taxonomy of Southeast Asian mammals and facilitating conservation planning using the bat fauna as a model system. Bats represent about 40% of the currently recognized mammalian species in the region and have been proposed as important indicators of the state of ecological communities for biodiversity assessments [22]. Specifically, we examine three main questions: (1) the extent to which currently recognized taxa can be uniquely identified using DNA barcodes; (2) the extent to which currently recognized species show deep genetic divides which may be suggestive of previously unrecognized species; and (3) whether DNA barcodes show evidence of geographic differentiation within widespread species suggestive of lineages that should be considered as separate units in conservation planning.

## Results

**Figure 1:**
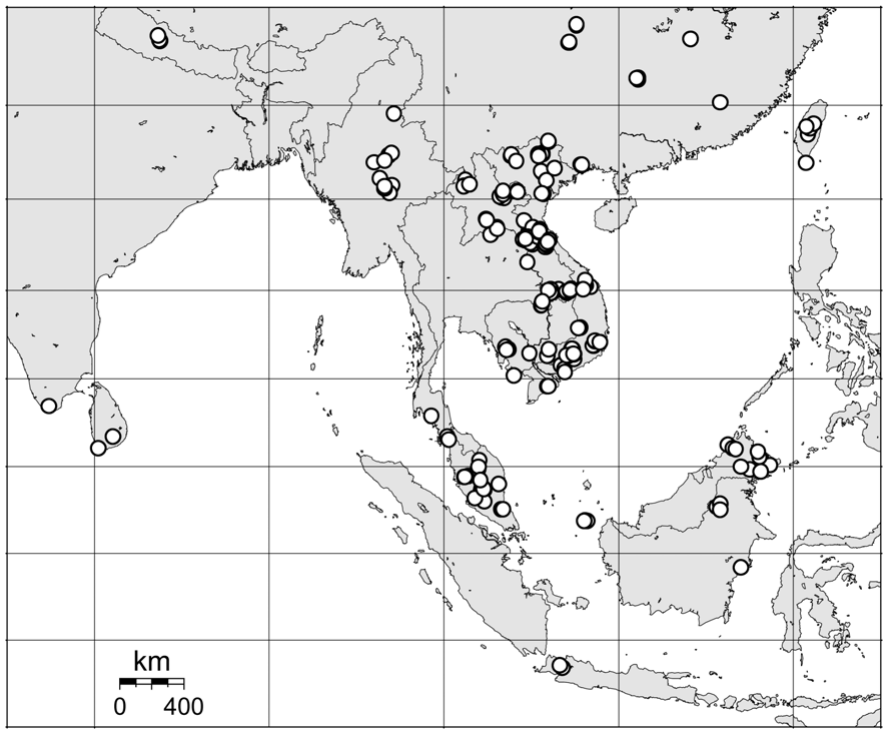
Distribution of collecting localities for the 1896 specimens analysed in this study. The majority of specimens came from Vietnam (665), Laos (561), southern China (279) and Malaysia (221) with smaller numbers from other countries. Map generated using the Online Map Creation Tool (http://www.aquarius.geomar.de/omc/)

We obtained DNA barcodes from 1896 specimens representing 157 morphologically distinct species from Asia, predominantly from southern China, Laos, Vietnam, peninsular Malaysia and Borneo (Figure 1). Of these, 142 species were assigned names based on currently published taxonomy, while an additional 15 species were recognized as morphologically distinct, but were either undescribed or could not be assigned appropriate names based on available reference material. Most species (133 out of 157) were represented by multiple specimens, with an average of 12 specimens per species; six species were represented by more than 50 specimens.

**Figure 2:**
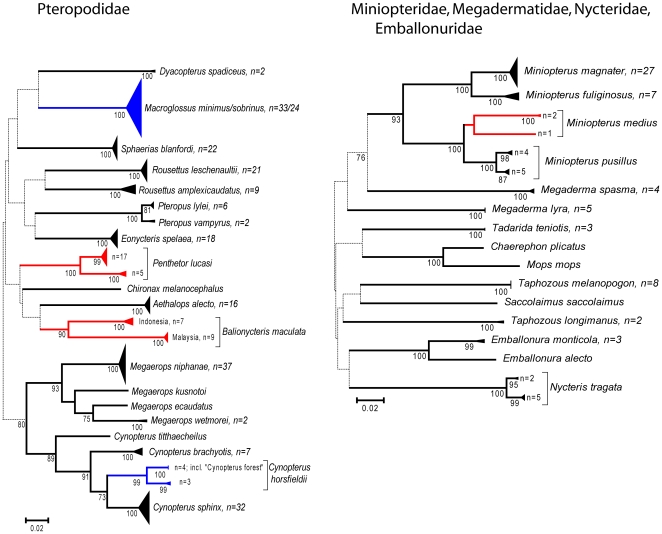
Neighbour-joining tree for bats in the families Pteropodidae, Miniopteridae, Megadermatidae, Nycteridae and Emballonuridae. Solid triangles represent clusters of multiple specimens, with the vertical dimension proportional to the number of specimens (shown as n = ), and the horizontal depth proportional to the genetic variation within that cluster. Blue indicates clusters of specimens that include more than one species that could not be resolved. Red indicates taxa with deep intra-specific divides that potentially represent distinct species. Numbers below joining branches indicated the level of bootstrap support for the branch — dotted lines indicate branching orders that were not strongly resolved (bootstrap support less than 70%).

**Figure 3:**
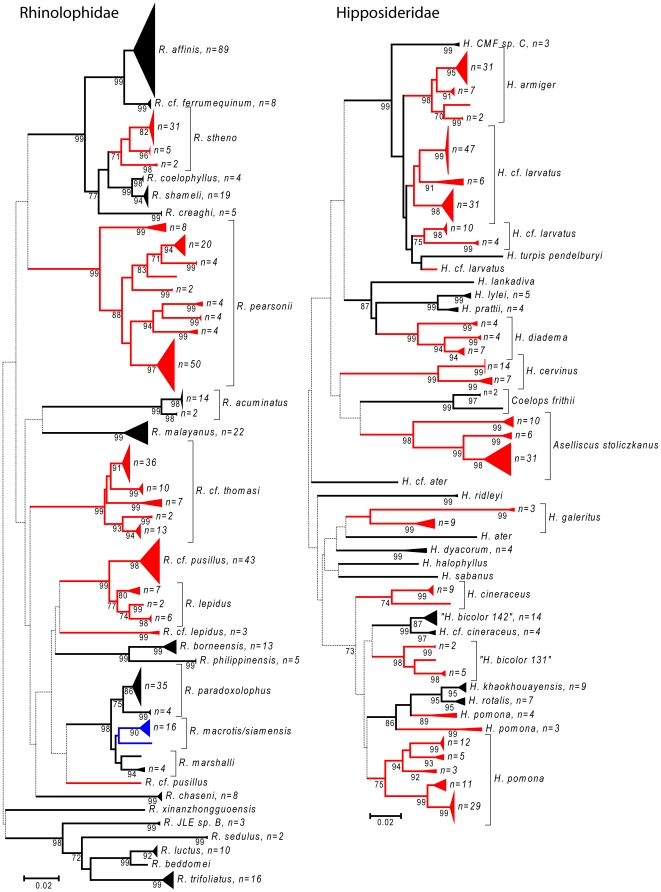
Neighbour-joining tree for bats in the families Rhinolophidae and Hipposideridae. Solid triangles represent clusters of multiple specimens, with the vertical dimension proportional to the number of specimens (shown as n = ), and the horizontal depth proportional to the genetic variation within that cluster. Blue indicates clusters of specimens that include more than one species that could not be resolved. Red indicates taxa with deep intra-specific divides that potentially represent distinct species. Numbers below joining branches indicated the level of bootstrap support for the branch — dotted lines indicate branching orders that were not strongly resolved (bootstrap support less than 70%).

**Figure 4:**
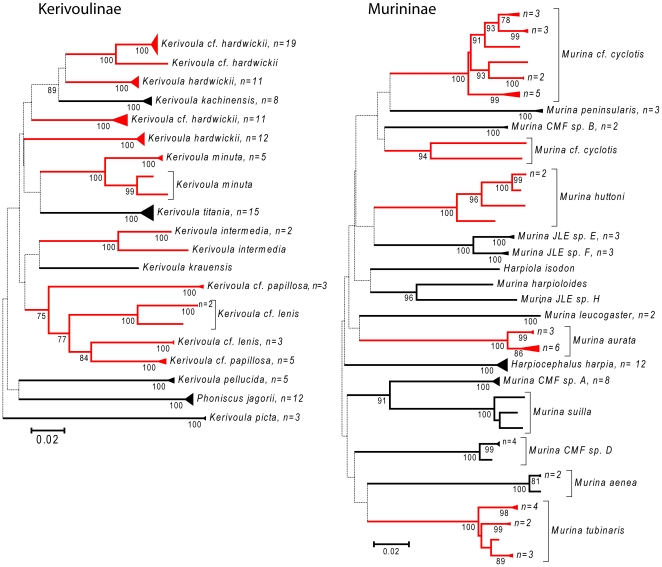
Neighbour-joining tree for bats in the subfamilies Kerivoulinae and Murininae of the family Vespertilionidae. Solid triangles represent clusters of multiple specimens, with the vertical dimension proportional to the number of specimens (shown as n = ), and the horizontal depth proportional to the genetic variation within that cluster. Red indicates taxa with deep intra-specific divides that potentially represent distinct species. Numbers below joining branches indicated the level of bootstrap support for the branch — dotted lines indicate branching orders that were not strongly resolved (bootstrap support less than 70%).

**Figure 5:**
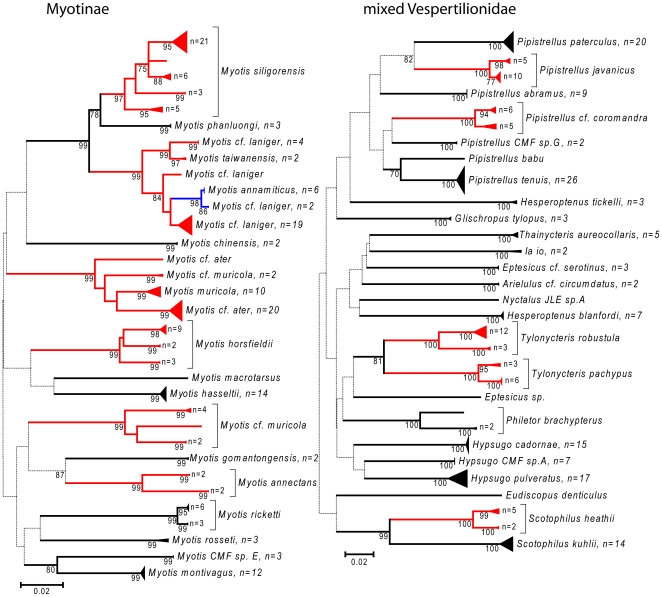
Neighbour-joining tree for bats in the subfamily family Vespertilionidae, including the subfamily Myotinae and mixed other subfamilies. Solid triangles represent clusters of multiple specimens, with the vertical dimension proportional to the number of specimens (shown as n = ), and the horizontal depth proportional to the genetic variation within that cluster. Blue indicates clusters of specimens that include more than one species that could not be resolved. Red indicates taxa with deep intra-specific divides that potentially represent distinct species. Numbers below joining branches indicated the level of bootstrap support for the branch — dotted lines indicate branching orders that were not strongly resolved (bootstrap support less than 70%).

**Figure 6:**
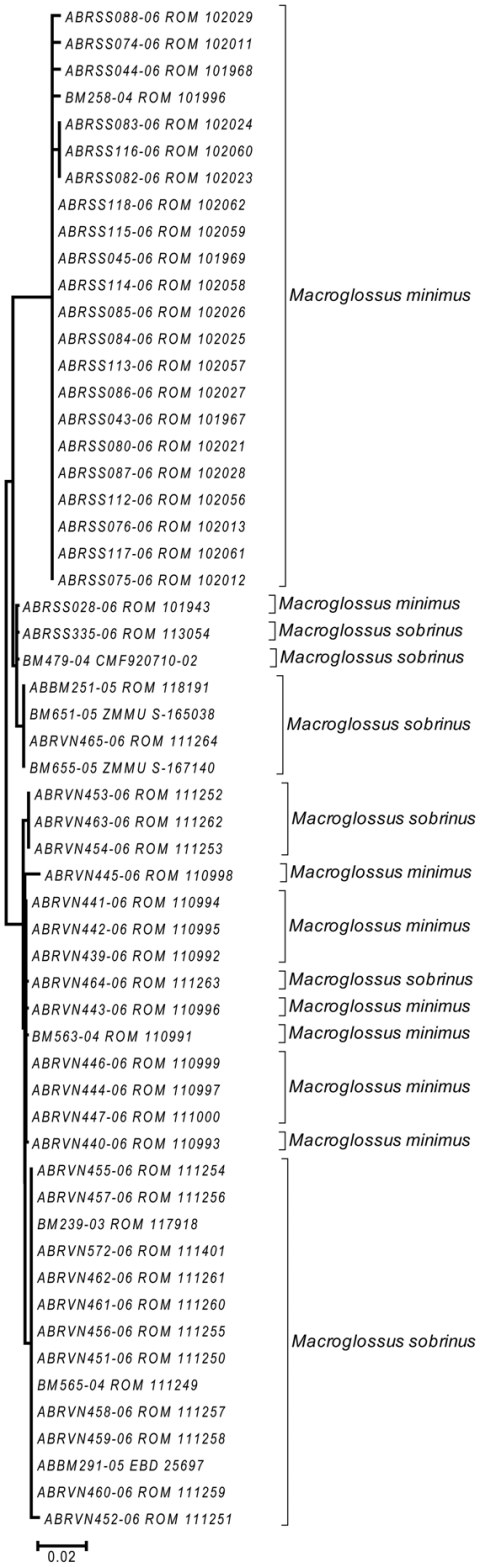
Detailed neighbour-joining tree for *Macroglossus sobrinus* and *M. minimus* showing individual specimens. Species identities are based on usually recognized morphological characters (Francis 2008), but do not correspond to genetic differences. The cluster at the top came from East Kalimantan, while the remainder were from Java, peninsular Malaysia, Thailand, Laos and Vietnam.

**Figure 7:**
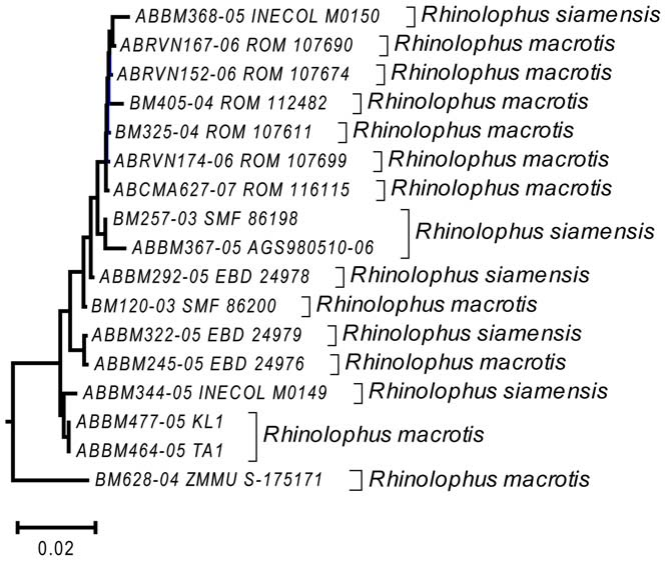
Detailed neighbour-joining tree for *Rhinolophus macrotis* and *R. siamensis* showing individual specimens. These two forms were readily separated by size and echolocation calls. The specimens came from Laos, Vietnam, Myanmar and southern China. The outlying specimen at the bottom is from southern Vietnam and may prove to represent something different.

**Figure 8:**
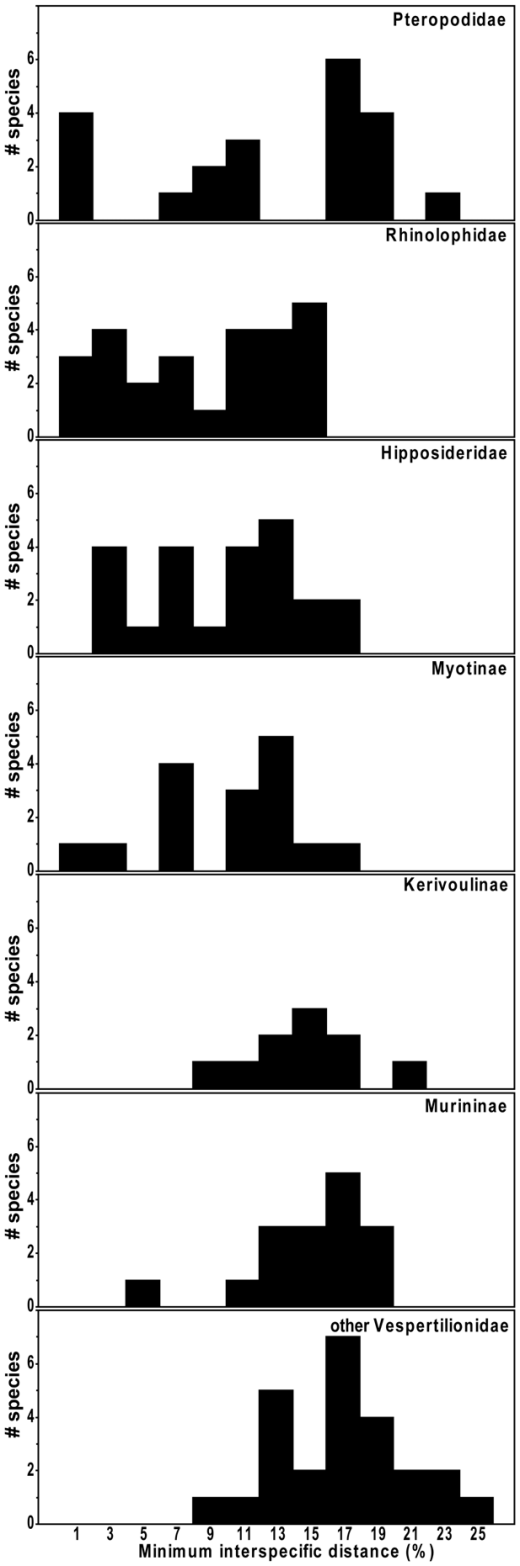
Distribution of nearest heterospecific neighbour distances by subfamily or family. Families with fewer than 5 species represented (Miniopteridae, Megadermidae, Nycteridae, Emballonuridae) are not shown.

Nearly all species could be uniquely identified based on DNA barcodes with the exception of four pairs of closely related congeneric species (see Figures 2, 3, 4, 5, 6 and 7). In a few additional cases, species were distinct, but were very similar genetically, such as *Pteropus vampyrus* and *P. lylei* which showed only 2% sequence divergence (Figure 2). However, in most cases, interspecific genetic distances were large, with the minimum genetic distance to the nearest species averaging 12.9% (SD 6.0%) and ranging up to 26% (Figure 8).

The four species groups that could not be clearly distinguished based on barcodes each had slightly different patterns of haplotype divergence. Specimens referred to *Macroglossus minimus* and *M. sobrinus* showed some geographic genetic structure, but this did not correspond with currently recognized species boundaries based on morphology (Figure 6). *Cynopterus horsfieldi* had three specimens with a diagnostic genotype, and two with a genotype that was 3.5% different (Figure 2), but the same as that of *C. brachyotis* “Forest” [23]. *Rhinolophus macrotis* and *R. siamensis* differed in size and echolocation frequencies but had low barcode variation which was not congruent with morphology (Figure 7). *Myotis annamiticus*, although morphologically distinct [24], differed by only 0.5% from its nearest neighbour and was nested within *M. laniger*, which showed intraspecific variation of up to 2.9% (Figure 5).

The amount of genetic differentiation among species varied among the 7 families or subfamilies for which we had more than 5 species represented (Figure 8). Mean minimum inter-specific distances ranged from a low of 8.6% (SE 1.0%) in Rhinolophidae up to 17.1% (SE 0.8%) in miscellaneous groups of Vespertilionidae. Among the Murininae and Kerivoulinae, which are each dominated by one genus (*Murina* and *Kerivoula* respectively), interspecific nearest neighbour distances were almost invariably very high, even between species that are morphologically very difficult to distinguish.

**Figure 9:**
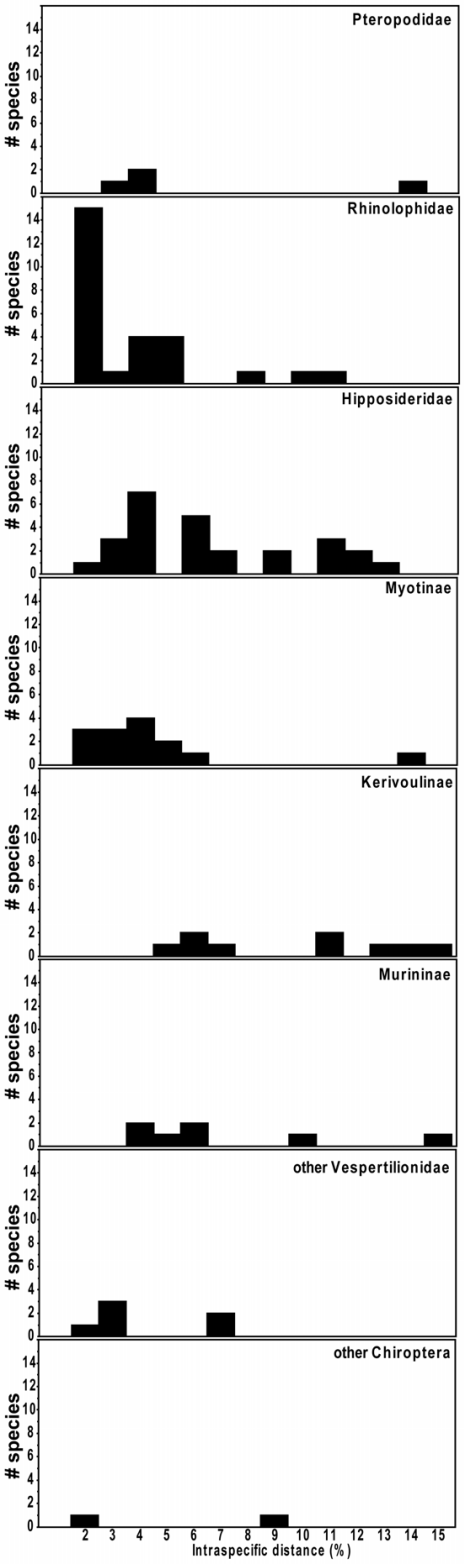
Distribution of nearest neighbour distances among genetically distinct clusters of individuals within species. Results are presented separately for each subfamily or family. The group “other Chiroptera” includes species in the families Miniopteridae, Megadermatidae, Nycteridae and Emballonuridae.

Of the 133 species with multiple specimens examined, 42 species, or about one third, had two or more barcode clusters differing by at least 2%. Some taxa had multiple clusters; one species (*R. pearsonii*) included nine lineages all differing from each other by more than 2%. The number of distinct haplogroups and their degree of genetic divergence within species differed among families (Figure 9), with families having the highest degree of interspecific variation also having high levels of intraspecific variation. Considering all species, we could recognize 95 additional haplogroups differing by more than 2%, of which nearly half differed by more than 5%.

**Figure 10:**
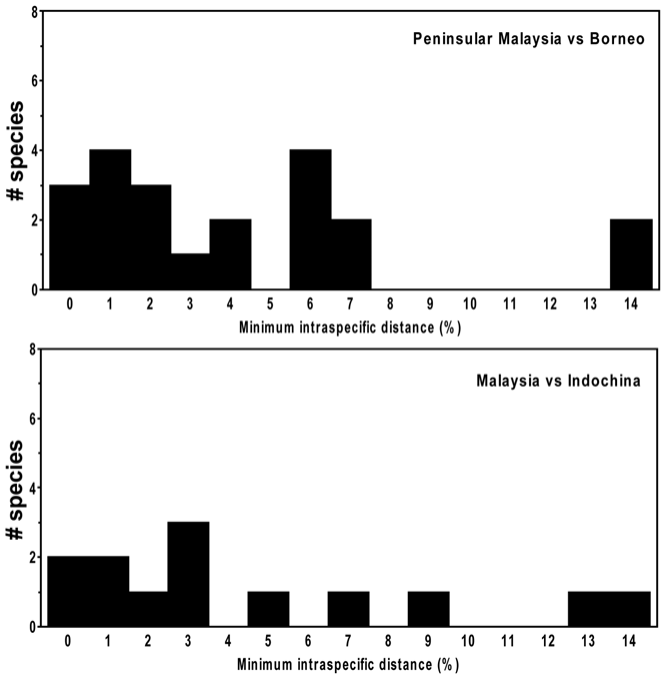
Distribution of nearest neighbour distances between members of the “same” species from disjunct geographic areas. The comparisons include 21 species from both peninsular Malaysia and Borneo, and 13 species from Malaysia (11 from Peninsular Malaysia and two from Borneo) and Indochina.

Most widespread species for which we had samples from multiple geographic areas showed substantial geographic variation in DNA barcodes (Figure 10). Of the 21 species for which we had samples from both peninsular Malaysia and Borneo, only three showed less than 1% genetic divergence between locations, while eight differed by more than 6%. Of the 13 species examined from both peninsular Malaysia (or Borneo) and Indochina, only two showed no divergence, while five differed by 5% or more.

## Discussion

Our analyses indicate that DNA barcodes are an effective tool for both differentiating and identifying species of bats in Southeast Asia. Although this study has only examined bats, early results from some of our team suggest that barcodes are similarly effective at differentiating other mammals in the region, including rodents and insectivores (C. M Francis, J. L. Eger, A. V. Borisenko, unpublished data). This suggests that DNA barcoding will enhance the effectiveness and efficiency of both conservation planning and research activities for all mammals in the region by assisting with species delineation and identification.

Our study has revealed that mammalian biodiversity in this region, at least measured in genetic terms, is much higher than previously recognized. Most widespread taxa showed substantial geographic variation in their barcode sequences, with populations from different regions such as peninsular Malaysia and Borneo often being genetically distinct (Figure 10). Although not all of the genetic divides that we detected necessarily represent new species, they do represent lineages with long histories of evolutionary independence, at least among maternal lineages. Furthermore, we only sampled part of the fauna that is shared among regions. For example, most of the approximately 100 bat species reported from peninsular Malaysia also occur in Borneo, but we only sampled 21 species from both areas. If those species are representative, we anticipate that two-thirds of the bat taxa shared by these areas will show at least 2% sequence divergence in COI, and one-third will differ by more than 6%; many of these likely represent distinct species.

High levels of genetic differentiation can also be anticipated among the many islands within the Philippine and Indonesian archipelagos, most of which we did not sample. Currently, many species are thought to be shared among numerous islands, but, assuming similar levels of biogeographic separation to the regions we did sample, we anticipate that many of those will prove to represent genetically distinct lineages. Similar levels of divergence may also occur among widely separated areas on the mainland, as noted by the results of our comparisons between peninsular Malaysia and Indochina. Sequencing tissue samples from these regions, either from existing or new collections, should be a high priority for understanding speciation in south-east Asia.

We also found several sympatric lineages showing deep genetic divergence and anticipate many more will be discovered with further sampling. Even allowing for the fact that some of these branches do not represent distinct species (see below), we suspect that bat species diversity in south-east Asia is at least twice that currently recognized.

This reassessment suggests a much higher level of endemism than currently recognized, a conclusion with significant implications for conservation planning. Adequate conservation of biodiversity in Southeast Asia requires protection for the complete suite of species within each geographic subregion, through a combination of protected areas and effective conservation in anthropogenic landscapes. Apparently widespread species are unlikely to be adequately protected by the designation of just one or a few reserves. Distinct biogeographic regions such as peninsular Malaysia, Borneo, and Indochina must be viewed as separate units for conservation planning. The same is likely to be true for many of the islands in Indonesia and the Philippines. Evidence of genetic differentiation in several species within Indochina suggests that it may be necessary to define areas of conservation importance at smaller scales, such as different ecoregions.

The observed high levels of genetic divergence suggest that, in addition to increased taxonomic effort to define species, ecological studies and field surveys are needed in each region to determine the ecological and conservation requirements of each species and any genetically divergent lineages. Genetically distinct populations, whether or not they are considered different species, are likely to have distinctive ecological requirements which need to be studied to ensure effective conservation planning.

Our data support the utility of DNA barcodes as a tool for field researchers carrying out faunal surveys [21] as well as ecological or behavioural studies. Despite the recent availability of comprehensive regional field guides (e.g., [2], [14]), reliable identification of many species of bats and other small mammals is challenging. For some species, their taxonomic identification can only be validated by careful examination of internal characters such as skull or baculum shape [2], [25]. By contrast, sufficient tissue for DNA analysis can be collected from a live mammal through a small biopsy (e.g., [26]) or via a blood sample with minimal adverse impact on the animal. When working in protected areas where collecting is not possible, or when carrying out behavioural studies of live animals, DNA barcodes recovered from biopsy samples will allow validation of identifications at a relatively low cost with a high degree of confidence.

The use of DNA barcodes as an identification tool by field workers requires the prior construction of a carefully validated reference database matching DNA barcodes to professionally curated specimens identified through traditional taxonomic work. Although our study has produced an initial dataset, most linked to museum vouchers, much additional work is needed to complete the database. We have not yet sampled all currently recognized species, and the results of our study suggest the likelihood of many undescribed taxa and further genetic variants to be discovered, especially in new geographic areas. When carrying out distributional surveys, especially in new areas, we recommend retaining a representative set of voucher specimens for deposit in a properly curated and publicly accessible collection because of the likely discovery of new taxa or genetically distinct populations. Tissue samples for molecular analysis should also be obtained from these specimens and preserved using appropriate protocols. DNA barcodes should then be promptly sequenced and published on shared international databases such as BOLD [27] to ensure the rapid sharing of knowledge about this diversity to aid in conservation planning.

DNA barcodes can also help taxonomists by facilitating comparison with other taxonomic material, even at a distance (e.g., [28], [29]). One of the challenges for a mammal taxonomist working in Southeast Asia is that most of the larger and reliably identified reference collections are scattered among museums, primarily in North America and Europe. Travel to those collections, or shipping specimens for comparison, is becoming increasingly difficult and expensive. In contrast, with ongoing improvements in technology, high quality sequences can be obtained cheaply from very small tissue samples. Moreover, the digital nature of genetic information makes DNA barcodes readily comparable through internationally accessible online data portals such as BOLD [27] or GenBank. Sequences in the BOLD database that are associated with specimen records linked to museum vouchers are the most valuable as reference material. By confirming the identification of specimens through DNA barcodes, local museums can establish reference collections that can serve as a basis for future research including the description of new species. DNA barcodes can also facilitate international collaboration. For example, Bates et al. [28] used them to confirm that specimens stored in different museums in Canada and Europe represented the same taxon which they subsequently described as a new species (*Kerivoula titania*).

Finally, DNA barcodes are a valuable tool for highlighting areas in need of further taxonomic research. Baker and Bradley [30] noted that divergent mtDNA sequences are often an indicator of unrecognized genetic species. We found 95 genetically distinct clusters differing by more than 2% from their nearest neighbours (Figure 9); a level which separated several pairs of morphologically distinct species (Figure 8). However, we agree with Baker and Bradley [30] that a simple threshold value, especially one based on a single gene, is not a sufficient basis for species recognition. While many of these clusters likely represent previously unrecognized taxa (especially the 15 that differ by more than 10% from their nearest neighbour), others may not, regardless of whether a biological or genetic species concept is adopted. A variety of processes including incomplete lineage sorting or introgression through a past hybridization event could lead to high levels of genetic variation within species [31], [32]. Female philopatry to breeding locations could lead to differentiation of mtDNA lines, even if there is extensive interbreeding and nuclear gene flow due to male dispersal. Variation in mtDNA can be retained for long periods if there is no selective pressure against it [32]. Nevertheless, substantial divergence in DNA barcodes can help to identify priority groups for further taxonomic study using other characters including morphology, behaviour (e.g., echolocation calls), or other genetic markers to determine which haplogroups do, in fact, represent distinct species.

Regardless of which characters are used to identify new species, DNA analyses supplement, but do not replace traditional morphological studies. Morphological examination of type specimens is still needed in most cases to determine whether the taxon already has a name. For example, if animals formerly regarded as conspecific are shown to represent two or more species, the original type must be examined to determine which of the newly proposed forms represents the original species name. In many cases, the types of closely related taxa must also be examined, including those currently considered as synonyms or subspecies. Early taxonomists working in Southeast Asia often coined names for different populations or even different colour morphs that were later synonymized; some of these may well prove to be valid taxa. Ideally, DNA barcodes should be obtained from all type material, and we urge researchers describing new taxa to ensure that properly preserved tissue samples and DNA barcodes are available for their type series, especially the holotype. Because most extant types were collected before the introduction of molecular techniques, many were preserved as dried skins, sometimes with added preservatives such as arsenic, while others were fixed in formalin before storage in alcohol. While methods for recovering sequences from old tissues are improving [33] and a minimalist (∼100 bp) barcode approach can be sufficient to link recently collected material to old types [34], [35], it remains difficult, time-consuming and expensive to extract DNA from older museum material, and success rates are low [36]. Furthermore, work with archival DNA requires a special laboratory setting and care to avoid contamination [37]. Finally, many museum curators remain reluctant to allow destructive tissue sampling of types for DNA extraction until analytic protocols are improved. As a consequence, DNA barcodes are not available for most mammal type specimens, so morphological comparisons remain the only available approach.

We conclude that DNA barcodes will greatly facilitate the challenge of properly describing and mapping biodiversity in Southeast Asia for the benefit of conservation. Such work is urgently needed because, despite evidence of high levels of genetic diversity within many species, conservationists and politicians still focus their effort around named species, as do data compendia such as the IUCN Red List [12]. Given the urgency for robust conservation actions within this region, where many habitats have already been lost, we hope that the use of DNA barcodes and public access of such information through Web portals will encourage the intensified taxonomic effort needed to describe and catalogue this diversity and ultimately to aid its protection.

## Materials and Methods

### Ethics Statement

All tissue samples came from specimens that had already been collected as part of other biodiversity studies which had been carried out with appropriate permissions from local authorities.

### Field Sampling

These biodiversity surveys were carried out at over 200 locations in South East Asia, mainly in southern China, Myanmar, Laos, Thailand, Vietnam, Cambodia, Malaysia and Indonesia between 1993 and 2006 (Figure 1). Much of the survey work was carried out by teams involving one or more of the authors of this paper, although some material was provided by additional researchers listed in the acknowledgements. Bats were trapped in the field using a variety of methods including mist nets, harp traps [38], and flap traps [39] for free-flying bats, as well as capture by hand or with small nets from roosts in caves, trees or buildings. Bats were measured and weighed and given a preliminary identification in the field. Tissue samples were largely taken from bats that were euthanized and preserved as museum specimens. Most tissue samples were heart, kidney, liver or muscle that was preserved in liquid nitrogen, 95–99% ethanol or in a DMSO solution [40]. Vouchers were prepared either as a dry skin and skeleton, or preserved in alcohol, usually after initial fixation in formalin. Most vouchers were later deposited in one of several museum collections as indicated in the relevant specimen record in the Barcode of Life Data (BOLD) systems (http://www.barcodinglife.org — see details below). A few tissue samples were obtained from wing punches [26] taken from live bats that were subsequently released. A small number of additional samples were taken from skin or muscle from museum specimens that had been collected up to 20 years earlier and preserved in 70% ethanol. However, few of these older preserved samples amplified successfully, and those that did often yielded only short sequences.

DNA extraction and sequencing success varied with the source of the tissue, the mode of preservation, and with the species group, but we did not track success rates due to changes in analytical protocols over the four years of this study. New primer cocktails were developed over the course of the study, improving sequencing success. In addition, accurate records were not always available on the tissue type or preservation methods used. As a result, for this paper we only consider samples that were successfully sequenced.

Bats were identified based on morphological criteria described in key taxonomic references including Corbet and Hill [4], Payne *et al*. [13], Bates and Harrison [25], Borissenko and Kruskop [22], Csorba *et al*. [41] and Francis[2], as well as primary literature reviewing or describing individual species. In most cases, taxonomy has been updated to match Simmons [42] except for species that have been described or recognized more recently or for which our own research indicates an alternative name is more appropriate. In most cases, specimens were initially identified in the field and then confirmed through subsequent examination of museum skulls and dental characters. In several instances, conflicts between DNA barcode results and initial identification of a specimen prompted its re-examination and a correction in the identification. We also detected several cases where tissue samples had been mixed up or mislabelled. In most cases the solution was easily deduced and the error was corrected. For example, if two representatives of morphologically distinct species collected at the same time had sequences that matched each other's species, we assumed they had been transposed and corrected the records. In a few other cases where an error seemed highly probable, but the cause could not be unambiguously determined, the data record was omitted from analysis.

### DNA extraction, PCR amplification, and DNA sequencing

Sequence analysis was carried out at the Canadian Centre for DNA Barcoding using standard high-throughput barcoding protocols [43]. Small pieces of tissue (approximately 1–2 mm^3^) were used for DNA extraction. Several methods were used throughout the duration of the project: Mammalian Genomic DNA Miniprep kit (Sigma-Aldrich), or in-house developed protocols Chelex-based ‘DryRelease’, silica-based ‘Silitom’ [43] and automated DNA extraction protocol on the Biomek FX© liquid handling station using 1.0 µm PALL glass fiber media filter plates [44].

A 652–657 base pair segment of COI was amplified using non-tailed or M13-tailed vertebrate primer cocktails [20], [44], [45]. In cases where we were not able to recover a full length barcode, the internal primer RonM [46] and its M13-tailed modification [21] was used. The 12.5 µl PCR reaction mixes included 6.25 µl of 10% trehalose, 2 µl of ultrapure water, 1.25 µl of 10X PCR buffer, 0.625 µl of MgCl_2_ (50 mM), 0.125 µl of each primer (0.01 mM), 0.0625 µl of dNTP mix (10 mM), 0.3125 U of Taq polymerase (New England Biolabs or Invitrogen), and 2.0 µl of DNA. PCR products were visualized on 2% pre-cast 96-well agarose gels (E-Gels©, Invitrogen) and the most intense products were selected for sequencing. Among samples that we included for analysis, 73% had full-length barcodes (650 or more base pairs), while only 2.5% had less than 400 base pairs (we excluded any sequence with less than 240 bp).

Products were labelled by using the BigDye© Terminator v.3.1 Cycle Sequencing Kit (Applied Biosystems, Inc.) as described in Hajibabaei *et al*. [43] and sequenced bidirectionally using an ABI 3730XL capillary sequencer following manufacturer's instructions.

Sequences are deposited in NCBI GenBank with accession numbers: HM540109 - HM542004. COI sequences, chromatogram trace files, and collateral specimen information are available in the Completed Projects section of BOLD in the project **Bats of Southeast Asia [BM]**.

### Tree building and genetic distance methods

Sequence data were managed using the Barcode of Life Data System (BOLD) [27] through its online interface at http://www.barcodinglife.org. Preliminary analyses were conducted using Neighbour-Joining (NJ) trees with a Kimura 2-parameter (K2P) distance model, as implemented with the Taxon ID tree function of BOLD. These were used to cross-reference the identifications inferred from sequences and morphology of voucher specimens. Once fully assembled, sequence data were downloaded from BOLD for further analyses. Analysis of genetic similarity was performed using MEGA version 4.0 [47], using the default parameters on the BOLD analytical module. All 657 sites (all codon positions and substitution types) were included in the analyses. Positions containing missing data were eliminated only in pairwise sequence comparisons (pairwise deletion option). Trees were built using the NJ algorithm with the K2P model. Branch support was assessed by bootstrapping with 500 replicates. To improve visualization of large data sets, we selectively compressed clusters of genetically similar specimens using the Compress/Expand function of the MEGA 4.0 Tree Explorer module.

For analysis of genetic distances within and among species, we first defined genetically distinct clusters within each species based on visual inspection of the NJ tree, using a combination of genetic distances and bootstrap support. We then calculated the mean genetic distance from each cluster to all other clusters both within and among species. We defined the minimum interspecific distance as the minimum distance from any individual within a species to its nearest neighbour in a different species within the same family (usually, but not necessarily, in the same genus). We also calculated distances among distinct haplogroups within the same species, particularly those between different geographic areas.
